# Integrating cross-omics research through FAIR Digital Objects with DataPLANT

**DOI:** 10.1515/jib-2025-0056

**Published:** 2026-06-17

**Authors:** Hannah Dörpholz, Rüdiger Simon, Björn Usadel, Angela Kranz

**Affiliations:** Institute of Bio- and Geosciences (IBG-4: Bioinformatics), CEPLAS, BioSC, Forschungszentrum Jülich, Wilhelm Johnen Straße, Jülich, Germany; Heinrich Heine University Düsseldorf, Faculty of Mathematics and Natural Sciences, Institute for Developmental Genetics, CEPLAS, Düsseldorf, Germany; Heinrich Heine University Düsseldorf, Faculty of Mathematics and Natural Sciences, Institute for Biological Data Science, CEPLAS, Düsseldorf, Germany

**Keywords:** FAIR data, NFDI, research data management, single cell transcriptomics

## Abstract

In plant sciences, single-cell and spatial transcriptomics generate large complex datasets which require structured metadata in order to make them interoperable and reproducible. In this work, we demonstrate how the Annotated Research Context (ARC) can be used for the management of such data using a barley single-cell RNA sequencing dataset integrated with spatial transcriptomics data. Experimental metadata was captured in ISA tables using ontology annotations to ensure machine readability and interpretability. The computational analyses were wrapped as Common Workflow Language (CWL) scripts to ensure reproducibility of the results and reusability of both the data and the analysis pipeline. The ARC links input materials unambiguously to the analysis results, allowing the research community to trace the entire investigation procedure. Our use case shows the benefits of using an ARC for structuring and annotating heterogeneous data, enabling comparative analyses across different datasets. While wrapping analysis scripts with CWL required some technical knowledge, the resulting standardized and reusable workflows outweigh this entry barrier. Parameter variations can be easily tested and results linked correctly without manual editing of the workflows themselves. Overall, this work highlights how using the ARC framework for single-cell datasets improves the FAIRness of the data and increases the reusability.

## Abbreviations

ARC: Annotated Research Context
CWL: Common Workflow Language
DOI: Digital Object Identifier
FAIR: Findable, Accessible, Interoperable, Reusable
GEO: Gene Expression Omnibus
HCA: Human Cell Atlas
ISA: Investigation – Study – Assay
MC: molecular cartography
minSCe: Minimum Information About a Single Cell Experiment
NFDI: National Research Data Infrastructure
RDM: Research Data Management
scRNAseq: single-cell RNA sequencing
SRA: Sequence Read Archive

## 1 Introduction

In the light of scientific and technological advancements, research data management (RDM) is becoming more and more important as the size and complexity of generated datasets continues to increase. Managing this data necessitates clear guidelines and standardization protocols across research facilities in order to make research data FAIR (Findable, Accessible, Interoperable, Reusable) [1]. By applying good RDM practices, data produced in a research group will remain interpretable and reusable long after the original researchers have left, underlining the long-term importance of data stewardship. The RDM life cycle typically encompasses the phases: *plan, collect, process, analyze, preserve, share* and *reuse* [2], 3]. In the planning stage, researchers are aided by data management plan tools to determine how the data of their project will be structured, documented, and maintained [4], 5]. During the data collection, processing and analysis, metadata is recorded in a structured format while adhering to community-accepted standards to ensure all experimental details are recorded, as well as all data post-processing workflows and analyses [6], 7]. Making use of ontologies in the metadata annotation ensures interoperability across different datasets and communities [8]. By publishing the generated data in trusted repositories, data preservation and provenance, as well as visibility and reusability are ensured [9].

While these RDM stages apply across different scientific domains, adoption by different communities often relies on additional characteristics of the individual research fields. In life sciences, the heterogeneity of high-throughput technologies and the complexity of biological systems generate a variety of different types of data which require specific metadata standards and infrastructures.

However, providing standardized metadata guidelines which are comprehensive enough to capture the relevant aspects of an experiment while simultaneously not overwhelming the scientist with their complexity is challenging. Such data can encompass phenotypic descriptions of organisms, sequencing information from DNA or RNA, protein abundances, metabolomic spectra and identification files, or data model representations, among many others. Different types of experiments require specifically tailored metadata, as well as sufficient metadata to link datasets across technologies for data integration. While there are multiple guidelines for reporting different types of data, such as the Minimum Information about any (X) Sequence (MIxS) [10] for sequencing information, or Minimum Information About a Plant Phenotyping Experiment (MIAPPE) [11] for plant phenotypic information, metadata guidelines for reporting single cell data are particularly sparse.

However (within the plant sciences), single-cell transcriptomics has become more and more popular since it enables the investigation of gene activity at cellular resolution [12], 13], allowing for in-depth analysis of the heterogeneity of different plant tissues, tracing developmental trajectories [14], 15], and understanding responses to environmental conditions [16], 17]. However, while these technologies were rapidly adopted by the plant community, the development of widely accepted metadata standards is still lacking, leading to challenges regarding integrating datasets across different studies. Though recent efforts have proposed best practices for plant single-cell transcriptomics in plants [18], 19], these have yet to be widely adopted by the community.

To address challenges such as inconsistent sample annotations, missing growth parameters or experimental conditions, and lack of data post-processing information, and to support sustainable RDM in plant sciences, the German National Research Data Infrastructure (NFDI) consortium [20] DataPLANT [21] was established. The NFDI association was founded to systematically manage scientific and research data across different domains. Within the life sciences domain, DataPLANT aims to provide tools, standards and infrastructure for sustainable research in the field of plant sciences to enable FAIR RDM practices across the plant science community. By focusing on the needs of the plant science community through active interactions between researchers and developers, DataPLANT bridges the gaps in metadata standardization, semantic annotation, and data processing reproducibility, facilitating data integration and reuse even of complex datasets.

In this work, we demonstrate how DataPLANT’s tools and infrastructure were applied to a dataset integrating single-cell RNA sequencing (scRNAseq) data with spatial transcriptomics data from barley, resulting in a barley transcriptome atlas.

## 2 Related works

Having outlined the general RDM challenges of single-cell transcriptomics in plants, we aim to summarize existing repositories, standards and guidelines in this section.

### 2.1 Data repositories

Multiple repositories provide access to scRNAseq data. These include most notably the Human Cell Atlas (HCA) [22], as well as the Sequence Read Archive (SRA) [23], the Gene Expression Omnibus (GEO) [24] and the European Nucleotide Archive (ENA) [25]. For all repositories, data can both be submitted and downloaded. However, data reusability varies between databases, based on their different metadata schema.

The HCA outlines a detailed metadata schema [26] and platforms for the metadata, with their tier 1 metadata detailing technical information required for data integration and the understanding of batch effects, tier 2 metadata detailing biological information about the samples, and cell annotation metadata detailing metadata on a per-cell level. As such, researchers can easily download and work with the count matrix of the datasets, as well as investigate the raw sequencing data.

In comparison, the SRA and the ENA only provide metadata regarding the samples, the sequencing library, and the sequencing procedure itself, as well as the raw sequencing data, but no derived data. In order to reuse these data, the appropriate pipeline for the outlined technology needs to be applied first in order to regenerate a gene expression matrix, which can be a daunting task without detailed knowledge of the experimental setup. This shortcoming can be overcome by uploading and reusing data from GEO. Here, both raw sequencing data, as well as derived data files such as the count matrices can be uploaded. Metadata encompasses a brief description of the experimental design, as well as description of the applied protocols such as treatments, plant growth, or extraction protocol, as well as information regarding the sequencing library and data processing can also be added. However, the degree of detail can vary greatly from dataset to dataset. In comparison, there are additional initiatives such as the Single Cell Expression Atlas, which contains curated scRNAseq data from any organism with metadata based on the recommendations in the previously reported Minimum Information about a Single Cell Experiment (minSCe) [18]. The count matrices for easy data reuse can be downloaded, as well as the raw sequencing data from cross-linked databases such as SRA or GEO. However, while highly curated, this database does not allow new data submissions.

### 2.2 Single-cell metadata guidelines

During the last few years, two notable guidelines for reporting scRNAseq data have been outlined [18], 19]. The Minimum Information about a Single Cell Experiment (minSCe) by Füllgrabe et al. mainly outlines metadata about the experimental design of a scRNAseq experiment, as well as the metadata required to reuse the raw sequencing data files, while Grones et al. focus on additional descriptions of the data processing, clustering validation, and data availability. During the BioHackathon Europe 2023, the work group “Ontologies for single-cell experiments” focused on reviewing minSCe schema while identifying metadata gaps for the description of plant-related experiments. These gaps mainly included the description of the protoplasting procedure, the removal of the plant cell walls, which is an essential step in the experimental procedure for plant cells [27], which is discussed in Grones et al. but missing from the list of recommended metadata terms.

In plant sciences, the recently formed The Plant Cell Atlas [28] community is combining efforts to provide a community resource describing the various plant cell types at high resolution. Still, the current data repository limitations requires researchers to publish their scRNAseq data to alternative repositories such as ENA for the raw sequencing data, or GEO, where sequencing files, as well as integral processed data such as count matrices and single cell data objects can be published as well, enabling easy reusability of the datasets.

Overall, it can be seen that there are still gaps in the infrastructure and metadata guidance in the plant single-cell community. Frameworks such as DataPLANT’s Annotated Research Context (ARC) [21], 29] provide a practical component to make project organisation FAIR. In the following sections, the RDM infrastructure that was utilized in our use case for managing transcriptomics datasets of barley is outlined.

### 2.3 RDM infrastructure

The ARC is a lightweight ISA-based (Investigation-Study-Assay) [30] FAIR Digital Object, complying with the Research Data Alliance’s FAIR Data Maturity Indicators [31], and is compatible with Research Object Crate (RO-Crate) [32]. The ARC concept was designed by DataPLANT as a digital object for organizing research data and metadata in a structured way, while fulfilling the FAIR principles outlined by Wilkinson et al. [1]. ARCs can be reused by other communities and platforms that rely on RO-Crate via conversion using DataPLANT’s tools [33]. The ARC root corresponds to the RO-Crate root, representing an ISA investigation. Other assays and studies are linked to that investigation with RO-Crate methods and follow their respective ISA RO-Crate profiles [34]. ARC runs and workflows can be converted based on the ARC CWL RO-Crate profile. As such, an entire ARC can be represented as an RO-Crate, making it compatible with other applications and workflows that are based on RO-Crate. In contrast, RO-Crate outlines multiple other profiles apart from ISA RO-Crate profiles and ARC CWL RO-Crate profiles as well, enabling easy (meta)data exchange between different communities and platforms.

ARC’s combine existing concepts from the ISA model and common workflow language (CWL) [35] to capture the majority of the research data life cycle, from experimental design and raw data generation to computational workflows and processed file outputs in a comprehensive, version-controlled research object. It was initially designed within the plant sciences community, but can be easily applied in other research fields. The aim of ARC is to allow researchers to collaboratively organize their analyses and make research data interpretable, reproducible and easily reusable.

To manage an ARC, the cross-platform application ARCitect [36] was developed, which enables users to set up and edit ARCs and synchronize them across platforms via the central PLANTdataHUB [21]. To annotate the experimental procedures with ontology terms, it integrates the Swate tool [37], which provides metadata templates in a familiar spreadsheet-based interface.

To cover the aspect of data storage, sharing and reusing of the research data life cycle, the PLANTdataHUB was set up, where ARCs can be stored and shared with selected collaborators. It provides an integrated publication service once an investigation is completed and the research data is meant to be shared with the public, where it will receive a referable DOI. Together with ARCitect and Swate, the PLANTdataHUB forms DataPLANT’s infrastructure for researchers to support them in managing their research data across the RDM life cycle.

## 3 Implementation

### 3.1 Experimental background

The previously presented concepts for RDM were applied in a cross-omics investigation on barley (*Hordeum vulgare*) [38]. In this study, transcriptional networks in barley shoot meristem development have been analyzed by integrating scRNAseq data and spatial high resolution single-molecule RNA-fluorescence *in situ* hybridization data through data imputation. This strategy leverages the strengths of both technologies, the broad transcriptome coverage in scRNAseq experiments, and the spatial resolution of *in situ* experiments, compensating for their individual limitations. The resulting database provides information on the expression of a large number of genes in each cell of a sectioned tissue.

**Figure 1:**
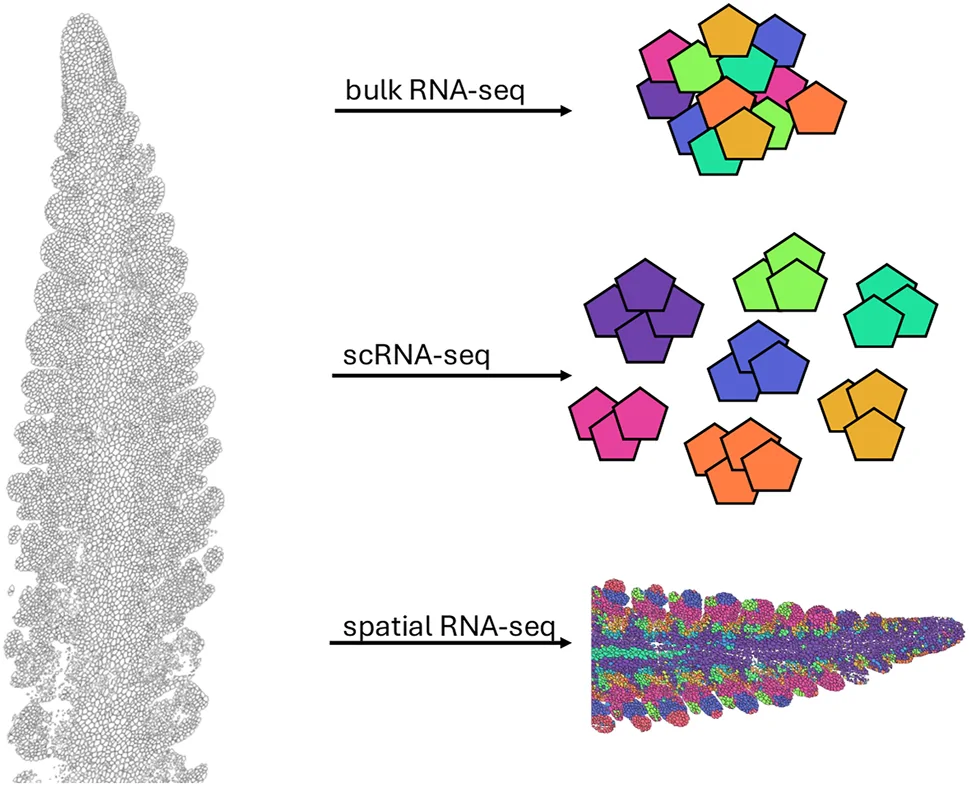
The three concepts of RNA sequencing are displayed. In bulk RNAseq, an averaged expression profile is obtained across all different cell types within the tissue. In scRNAseq, gene expression can be profiled on a cellular level and different cell types can be distinguished based on their expression. In spatial RNAseq, no tissue dissociation is performed, and gene expression can be profiled on a cellular basis while simultaneously preserving information about the cell’s spatial context.

Both methods were applied to *H. vulgare* in different developmental stages of spikelet development by Demesa-Arevalo et al. Instead of profiling gene expression on the tissue level, as is done in bulk RNAseq, scRNAseq using BD Rhapsody technologies and Resolve Biosciences’ Molecular Cartography (MC) were applied for single-cell profiling (Figure 1). In BD Rhapsody, the entire transcriptome of the cells can be captured, by isolating the individual cells, barcoding their RNA molecules, and sequencing the prepared library, allowing for the reads to be mapped back to their original cells. In contrast, MC provides spatial context by directly measuring expression of up to 100 genes in tissue sections, using indirect detection of gene-specific and barcoded probes in multiple rounds of imaging to record the RNA locations within the intact tissue. While the detailed computational strategies and biological findings are presented elsewhere [38], we focus on the structured documentation of the sample preparation, the experimental design, and the data analysis. This multi-omic approach, based on two technologies which capture different dimensions of the transcriptome, requires capturing different metadata to ensure reproducibility, interoperability, and future reusability of the generated data. In the following section we demonstrate how we could achieve (meta)data FAIRness using the ARC framework.

### 3.2 ARC in practice

**Figure 2:**
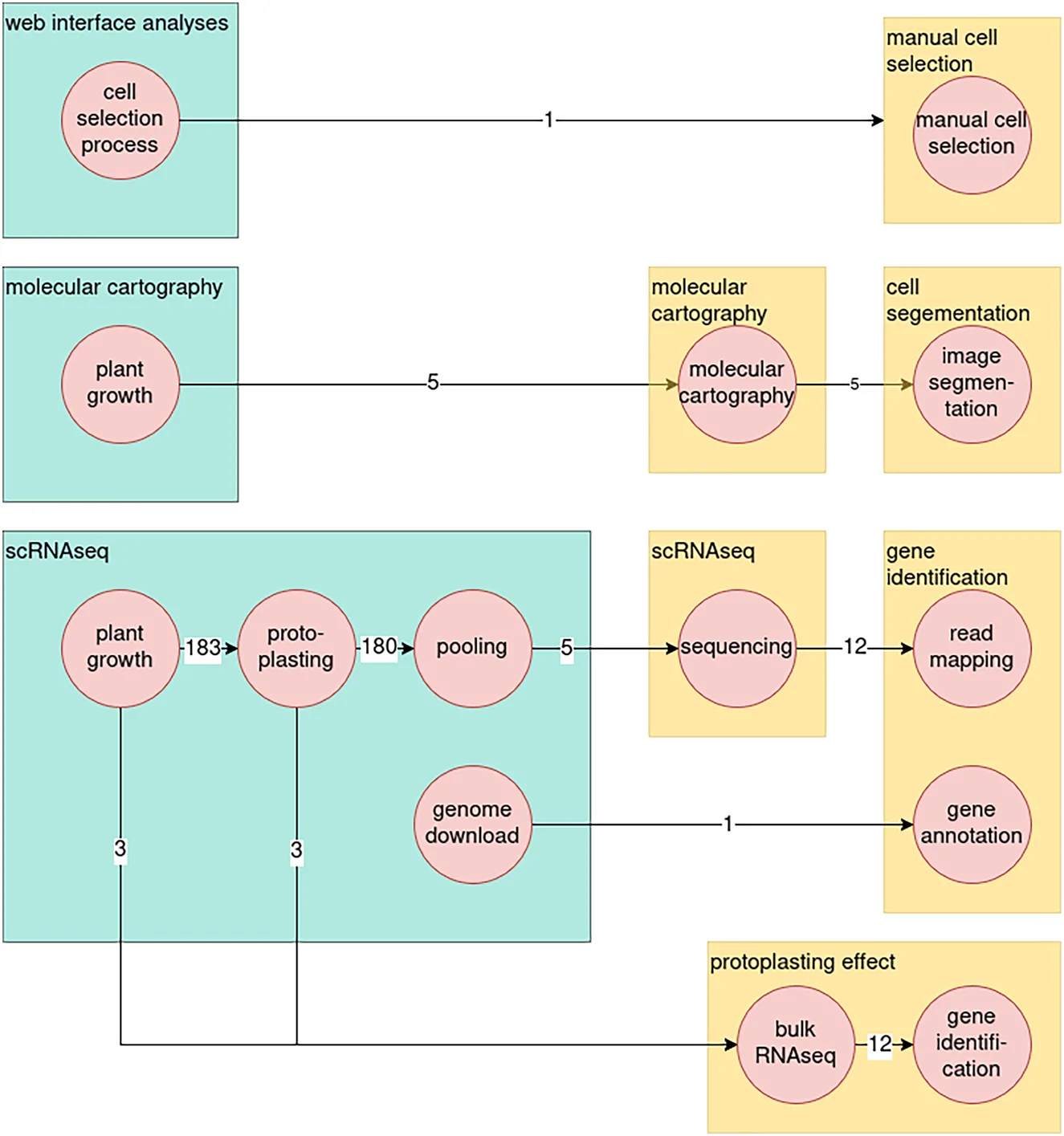
Relationships between assays and studies of the barley ARC. The ARC comprises three studies (indicated in green), one for the scRNAseq experiment, one for the MC experiment, and one for the web interface analysis. The assays (indicated in yellow) performed during the investigation are linked to the three studies, with three assays being linked to the scRNAseq study, two assays being linked to the MC study, and one assay being linked to the web interface analysis study. Arrows with numbers indicate the number of output materials from the individual processing steps (indicated in red) which serve as input materials for the next processing step.

To capture all (meta)data from the barley *investigation*, we created a new ARC (https://doi.org/10.60534/zfdth-2g147). At the top level, contact persons and their roles in the investigation, as well as the public preprint and a short description are outlined. With the experimental setup in mind, the information was split into three *studies* and six *assays* (Figure 2).

The first study outlines the sample processing for the scRNAseq experiment. It consists of four metadata tables which describe different steps of the sample preparation. The first sheet describes the plant growth, making use of the DataPLANT template “Plant growth conditions”, with additional modifications to account for a pre-germination step. The table outlines important characteristics such as organism, cultivar, age, day and night temperatures, and light regimen, as is outlined in the minimum information recommendations of the minSCe and from Grones et al. For each seed that was grown, one row was added to the table to track its subsequent processing. During this process we noticed ontological gaps regarding data for single-cell investigations. Specific terminology is not always available, which inspired us to start developing an ontology for scRNAseq studies, which both reuses the existing vocabulary, while simultaneously covering additional aspects relevant in single-cell studies in plants, keeping the existing metadata recommendations in mind.

The second table describes which of the samples from the plants were subject to the protoplasting procedure, where the cell walls were stripped from the cells, with detailed information about involved buffers and cell wall digestion enzymes. While this metadata is not explicitly outlined, it can be considered as part of the tissue dissociation process which is recommended information to record in both metadata schema.

The third table outlines how the individual samples were pooled for the subsequent analyses. Procedures like this are indicated by having multiple input samples point to the same output sample.

The fourth and last table provides information about the reference genome which would be used in the subsequent read mapping, under which DOI the resource is available, and it describes that it is located within the ARC in this studies’ *resources* directory.

The second study details sample processing for the MC experiment. It contains the single metadata table about the plant growth. Here, one metadata row describes one of the individual sections that were cut on the tissue.

The third study describes an intermediate step in the data analysis, in which cell subsets were selected using a web interface.

In total, six experimental *assays* were performed, three of which are linked to the sample material described in the scRNAseq study, and two are linked to the sample material described in the MC study. The last assay is linked to the web interface cell selection study. Using the output materials from the plant growth processing step of the scRNAseq study and from the protoplasting processing step, bulk RNAseq was performed to be able to analyze the effect of the protoplasting procedure on the gene expression. Three samples from each study processing step were used for this procedure (Figure 2) by following the information in the output and input columns in the metadata sheets, making it easy to understand the sample processing pipeline. The metadata in this bulk RNAseq assay describes the library preparation and the sequencing platform, as well as which output sequencing files were used for the identification of the genes affected by the protoplasting procedure.

Most of the samples described in the scRNAseq study were used for scRNAseq sequencing, with 180 sample outputs from the protoplasting procedure being pooled into 5 input samples for the sequencing. Here, the metadata outlines the number of input cells used, the library preparation procedure, and the sequencing instrument, resulting in sequencing raw data files.

The last scRNAseq assay describes how the BD Rhapsody analysis pipeline was run, which uses the sequencing files as input and maps them against the reference genome, which is described in further detail including genome name and genome version. In addition, the genome was annotated with Mercator4 [39], which is described in the last metadata table.

All metadata annotations were performed using primarily ontology terms from relevant ontologies including as OBI (Ontology for Biomedical Investigations) [40], NCBITaxon (NCBI organismal classification) [41], NCIT (NCI Thesaurus OBO Edition) [42], PO (Plant Ontology) [43], 44], BAO (BioAssay Ontology) [45], and GENEPIO (Genomic Epidemiology Ontology) [46], as well as DataPLANT’s own DPBO (DataPLANT biology ontology) [47]. This makes the metadata not only understandable, but also machine-readable, improving the overall interoperability of the results in the investigation.

With the experimental metadata structured in the ARC, we then linked the raw data outputs to the subsequent computational analyses through modular workflows.

### 3.3 Integration and analysis

After structuring the experimental metadata in the ARC, we then continued to integrate and analyze the generated raw data using CWL workflows (https://doi.org/10.60534/zfdth-2g147). In order to integrate the data from the two different methodologies, multiple different workflow steps needed to be applied to the data. To demonstrate the benefit of using an ARC with the underlying CWL structure for this, we will focus on outlining the procedure for a subset of our analysis pipeline.

The major two steps include integrating the scRNAseq data with the MC data, and the gene expression imputation, where expression of the genes not measured in the MC experiment is computationally determined, based on the gene expression of equivalent cells in the scRNAseq dataset. In order to provide a structured overview, each step of the processes was divided into smaller modules which can be easily re-run and tested without having to run the entire computational pipeline if only a few parameters should be tweaked and tested.

The data integration can be split into 5 steps: count matrix pre-processing, count matrix filtering, quality control, data integration, and clustering. While file pre-processing and filtering was done in *Python*, the subsequent analyses were performed with the *R* package *Seurat* [48]. *Seurat* provides methods for exploring scRNAseq data in an easy to apply and interpret manner. Here, we demonstrate how the developed workflows were integrated into the ARC structure and made executable with CWL exemplary for the data integration.

**Figure 3:**
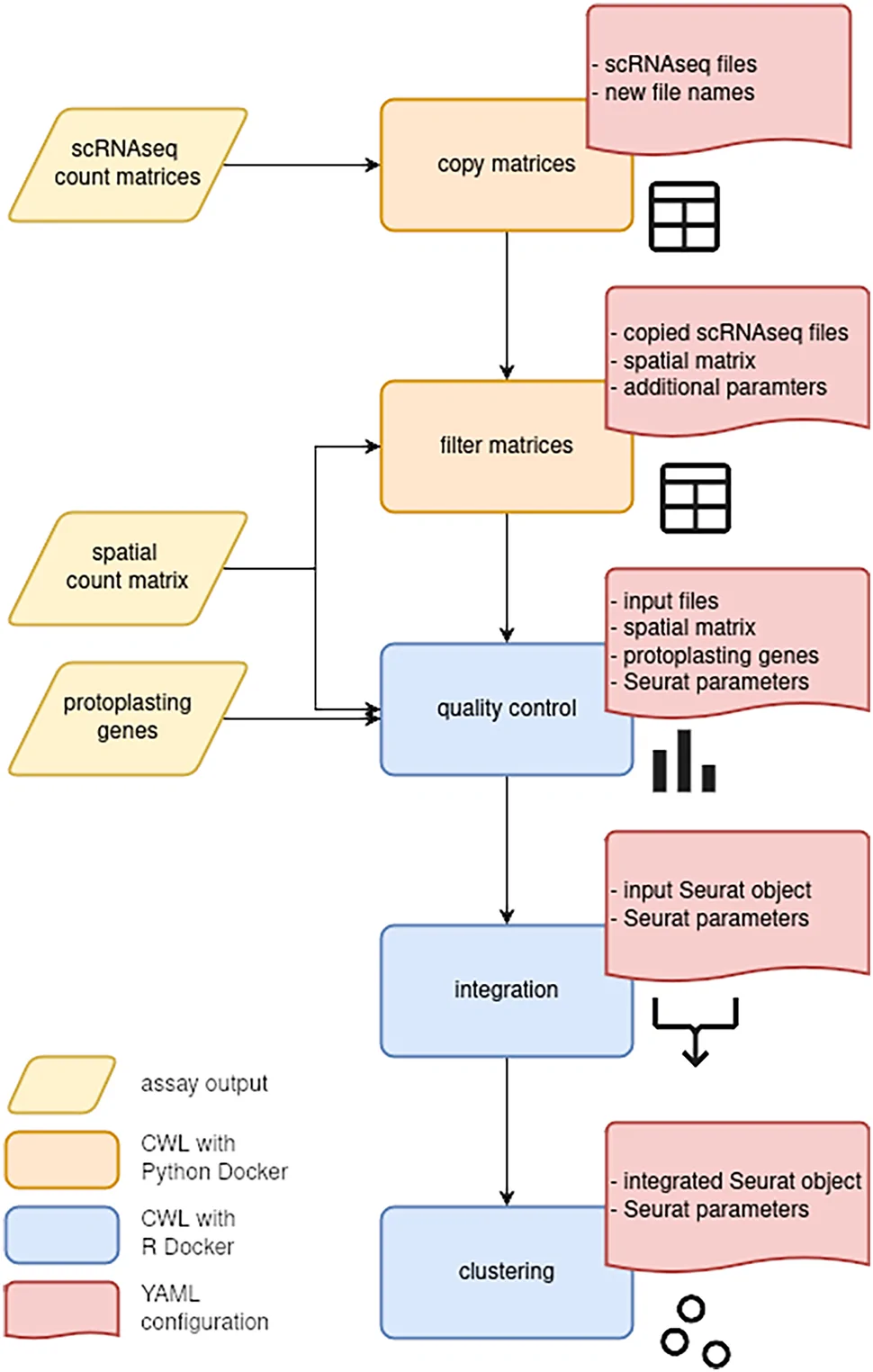
Detailed overview of the interactions between raw data outputs in assays (yellow parallelograms), analysis scripts in workflows (blue/orange squares), and workflow configurations in runs (red) for the initial data pre-processing and data integration with Seurat.

First, file pre-processing was performed using the scRNAseq data outputs from the sequencing with BD Rhapsody single cell technology (Figure 3). These consisted of three count matrices for three biological replicates as *csv* files containing information about the number of gene transcripts per cell. To ensure easier interpretability during the analysis, files were copied and renamed before any subsequent workflow steps.

For the downstream analyses with *Seurat* the matrices had to be filtered to exclude all genes from the three scRNAseq count matrices which were not measured in the spatial experiment, resulting in three highly reduced count matrices containing the same set of genes. These three filtered scRNAseq matrices, as well as the spatial matrix could then be used as inputs for the analysis with *Seurat*.

The *Seurat* analysis was split into three different steps, the initial quality control of the data, the integration of all four datasets, and the clustering of the integrated datasets.

All five of the analysis process steps were set up to work with CWL within the ARC structure. Each step is defined by (a) a CWL tool script, (b) a *Python* or *R* processing script, and (c) a YAML configuration file. The CWL script wraps around the processing script and defines how to run the processing script, which parameters and parameter formats are required for execution, and which resources are necessary, such as Docker containers, or a specific amount of RAM.

The processing scripts make use of the *argparse* package which is available for both *Python* and *R* in order to read the run parameters from the command line and use the information for adjusting the analysis procedure.

Lastly, the YAML configuration contains values for all arguments described in the CWL file, which are passed to the command line once the CWL tool is run. Here, in addition to run parameters, the input and output file locations can be specified.

All of the described CWL tools were then chained together into a CWL workflow which indicates the process order of the individual CWL tools and handles dependencies between workflows.

The described structure of the workflows enables a clear interpretability of the processing and unambiguously links final outputs (e.g. outputs used for a publication) back to the raw data files, and via the ISA metadata worksheets back to the original input materials used in the study. This linkage allows the researcher to clearly understand their own experiments and computational analyses, and helps identify weaknesses in their pipeline. By setting up the computational analysis in a modular way, errors and irregularities in intermediate and final output files can be easily narrowed down, and individual pipeline steps can be adjusted by creating new YAML configuration files with adjusted parameters to re-run individual steps. An additional layer of validation by CWL, which validates the general input formats of the YAML configurations against the requirements specified in the CWL files, aids in avoiding mistakes in the YAML configuration, such as trying to input a number where a string is required. Furthermore, the need of local installations for analysis tools can be avoided by providing Docker containers for the individual pipeline steps, allowing other scientists to easily reuse both the entire workflow pipeline, as well as individual steps with minimal effort.

After integrating the datasets based on the shared genes, gene expression imputation was performed [26]. Briefly, for each cell in the spatial dataset, the most similar cells in the scRNAseq dataset were identified based on their cosine similarity. Using the similarity as weights, an average gene expression was calculated across the most similar cells and assigned to the cell from the spatial dataset. This way, gene expression information which was not initially measured in the spatial dataset could be inferred using the complementary scRNAseq dataset. The resulting information was then displayed in an online transcriptome atlas. Here, multiple tissue sections can be explored, along with their gene expression information and gene annotations, cell cluster information, and information about gene up- and downregulation.

### 3.4 Data visualization

During the barley investigation of Demesa-Arevalo et al., a webpage was set up where the data are displayed. The BARVISTA (**Bar**ley **V**irtual ***i*** *n* ***s*** *itu* **t**ranscriptome **a**tlas) provides information on five different sections of barley, including three sections of the barley inflorescence meristem, one section of the barley vegetative shoot apical meristem, and one section of a mutant inflorescence meristem. The webpage allows researchers to view the different identified cell types, explore the gene expression of their genes of interest, and gain information about gene up- and downregulation, all within the spatial context of the cells. This enables the further study of specific regions of the barley inflorescence during development by allowing users to select individual cells and download the corresponding gene counts to use for their subsequent analyses. Furthermore, the webpage can be easily extended with data for additional sections such as different developmental stages or different tissues. Using the same framework, data from different plants could be displayed as well, with the possibility of generating a three-dimensional data presentation, reconstructed from serial tissue sections and gene expression imputation.

## 4 Discussion

We presented how the ARC framework can be applied to a multi-omic investigation in order to collect and structure heterogeneous metadata and data, as well as computational workflows, in an easily interpretable, reproducible and reusable way. Complementing the biological interpretation of the data, recording relevant metadata is just as important to be able to exploit the full potential of the data, both within the original investigation, as well as during data reuse at a later stage. As such, we aimed to make each step of the investigation as transparent and as FAIR as possible. This could be achieved using the ARC framework, which structures (meta)data along ISA tables using ontology annotations for experimental processes, and CWL workflows for computational processes, making the information of an investigation machine-readable and interpretable. Additionally, data provenance can be tracked by linking the initial input materials to the generated raw data outputs, across intermediate analysis files to the final computational outputs. This aids both the researcher performing the analysis to easily follow their own experiments once they plan to publish their findings, and other researchers who want to reuse the data at a later stage for their own analyses. Having clearly annotated, machine-readable process steps allows for an easier integration of the data, as well as meta-analyses across multiple well-annotated datasets. Furthermore, being able to work collaboratively on ARCs ensures a good information flow of all experimental and computational procedures between partners, allowing all participants an increased understanding of all aspects of the project. Another benefit of using the ARC framework is the semantic annotation of metadata with ontology terms. Making use of defined vocabulary avoids ambiguous interpretation of descriptions and enables a homogeneous usage of terms across different labs who might annotate information differently, such as using either *H. vulgare, Hv* or barley for the organism description.

Still, there are some limitations when it comes to using ARC as the framework for RDM as well. Recording experiment-specific metadata in table format provides a familiar and accessible environment for most plant scientists who are already used to working with spreadsheets to document their experiments. In contrast, wrapping computational processes in CWL and YAML requires some technical knowledge, which can be daunting for researchers without a programming background. Without much programming background, the entry barriers can prove daunting. Still, the long-term advantages of wrapping all computational processes in standardized and reusable workflows outweighs the initial effort required to learn about CWL, and we hope that our example presented here can be helpful in lowering the barriers of workflow development. Furthermore, particularly emerging technology fields such as spatial transcriptomics are currently not well-represented in ontologies. These gaps can slow down the metadata annotation process and force researchers to fall back to free-text annotations rather than ontology annotations. This is mitigated to some degree by DataPLANT’s own DPBO, which serves as a bridge between researchers missing specific metadata terms, and existing ontologies not yet covering the terms themselves. New terms can be easily requested via GitHub, allowing researchers to use specific vocabulary even if it is not yet covered by established ontologies.

Overall, we could show that ARC offers a versatile framework which can be applied to different types of investigations, although it is not a universal solution for every research question. However, it is a powerful aid for collecting structured metadata, provides data provenance, and enables reproducible workflows. More importantly, ARC is more than just a local solution for the German NFDI community [20]. Since ARCs adopt the RO-Crate framework, they are at the same time interoperable with existing international research data management infrastructures which use RO-Crate. As such, ARCs can be aligned with other initiatives at the European level, such as the European Open Science Cloud (EOSC) [49], and integrated into other frameworks beyond plant sciences. This interoperability at the international level ensures that the data of each ARC can have a long-term impact on the respective research community and enable data exchange across national boundaries.

Another promising advancement is the SciWIn tool [50] from the FAIRagro NFDI consortium [51]. This tool aids researchers in the creation of CWL tool scripts that wrap around the existing analysis scripts, and chains them together into a workflow. This reduces the technical entry barrier for researchers who are not familiar with workflow languages and CWL in particular, making it easier to integrate their own computational pipeline into the ARC. This way, SciWIn can directly complement DataPLANT’s RDM infrastructure and aid in the FAIRification process of the computational analyses.

In the near future, an integration of ARC with Galaxy is planned. Galaxy is already a widely adopted platform for running computational analyses which can be run both via the web-based interface, or through the command line [52]. As such, researchers will be able to benefit from both the structured metadata of an ARC, as well as the option to run their analysis in the familiar Galaxy environment. Going further than Galaxy, there is also a community demand for the support of the workflow languages Nextflow [53] and Snakemake [54]. While there is currently no support for these languages planned, the modularity of the ARC means that they could be incorporated in the future.

Additionally, the ARC format could be used to support downstream applications, such as visualization tools similar to the webpage generated for the barley data. This could facilitate both maintenance of the application, as well as the addition of new data by directly linking researchers and developers, allowing for a closer communication and interaction to address the needs from both sides at one single entry point.

In the future, there is much potential for using ARCs to capture single-cell data. For one, the clear metadata structure and machine-readable ontology annotations allow for easier data integration and reuse across different investigations, allowing for comparative analyses and meta-studies without extensive additional manual curation, thus increasing the value of the individual datasets. Similarly, by further aligning metadata collection guidelines and providing specific templates, ARCs can be closely aligned to public databases such as ENA or GEO, especially for the submission of single-cell data, ensuring that researchers capture both all relevant information to fully describe their dataset, as well as all information required by different repositories. This way, all data can be preserved in the respective community-supported databases, while links between data are still preserved within the ARC itself. To increase the adoption of ARC as an RDM framework, further focus should be put on lowering the technical barriers for building standardized workflows for the average user. Overall, we could demonstrate how ARCs can be applied to multi-omics investigations in plant sciences to improve dataset FAIRness and highlight that both the original research team, as well as external parties who want to reuse the data can benefit from the clear, unambiguous (meta)data annotations.

## References

[1] Wilkinson MD, Dumontier M, Aalbersberg IJ, Appleton G, Axton M, Baak A (2016). The FAIR Guiding Principles for scientific data management and stewardship. Sci Data.

[2] Whyte A, Tedds J (2011). Making the case for research data management.

[3] Cox AM, Pinfield S (2013). Research data management and libraries: current activities and future priorities. J Libr Inf Sci.

[4] Zhou XR, Beier S, Brilhaus D, Martins Rodrigues C, Mühlhaus T, von Suchodoletz D (2023). DataPLAN: a web-based data management plan generator for the plant sciences. Data (Basel).

[5] Gajbe SB, Tiwari A, Gopalji, Singh RK (2021). Evaluation and analysis of data management plan tools: a parametric approach. Inf Process Manag.

[6] Sansone SA, Rocca-Serra P, Field D, Maguire E, Taylor C, Hofmann O (2012). Toward interoperable bioscience data. Nat Genet.

[7] Rocca-Serra P, Sansone SA (2019). Experiment design driven FAIRification of omics data matrices, an exemplar. Sci Data.

[8] Riquelme-García A, Mulero-Hernández J, Fernández-Breis JT (2025). Annotation of biological samples data to standard ontologies with support from large language models. Comput Struct Biotechnol J.

[9] Qiu VY, Tse AWC Research trends and emerging themes of data repository for research data management services in higher education: a bibliometric analysis. ..

[10] Yilmaz P, Kottmann R, Field D, Knight R, Cole JR, Amaral-Zettler L (2011). Minimum information about a marker gene sequence (MIMARKS) and minimum information about any (x) sequence (MIxS) specifications. Nat Biotechnol.

[11] Ćwiek-Kupczyńska H, Altmann T, Arend D, Arnaud E, Chen D, Cornut G (2016). Measures for interoperability of phenotypic data: minimum information requirements and formatting. Plant Methods.

[12] Marand AP, Jiang L, Gomez-Cano F, Minow MAA, Zhang X, Mendieta JP (2025). The genetic architecture of cell type–specific cis regulation in maize. Science.

[13] Ke Y, Pujol V, Staut J, Pollaris L, Seurinck R, Eekhout T (2025). A single-cell and spatial wheat root atlas with cross-species annotations delineates conserved tissue-specific marker genes and regulators. Cell Rep.

[14] Yin R, Chen R, Xia K, Xu X (2024). A single-cell transcriptome atlas reveals the trajectory of early cell fate transition during callus induction in *Arabidopsis*. Plant Commun.

[15] Sun Y, Han Y, Sheng K, Yang P, Cao Y, Li H (2023). Single-cell transcriptomic analysis reveals the developmental trajectory and transcriptional regulatory networks of pigment glands in *Gossypium Bickii*. Mol Plant.

[16] Tang B, Feng L, Hulin MT, Ding P, Ma W (2023). Cell-type-specific responses to fungal infection in plants revealed by single-cell transcriptomics. Cell Host Microbe.

[17] Tenorio Berrío R, Dubois M (2024). Single-cell transcriptomics reveals heterogeneity in plant responses to the environment: a focus on biotic and abiotic interactions. J Exp Bot.

[18] Füllgrabe A, George N, Green M, Nejad P, Aronow B, Fexova SK (2020). Guidelines for reporting single-cell RNA-seq experiments. Nat Biotechnol.

[19] Grones C, Eekhout T, Shi D, Neumann M, Berg LS, Ke Y (2024). Best practices for the execution, analysis, and data storage of plant single-cell/nucleus transcriptomics. Plant Cell.

[20] Nationale Forschunsdaten Infratruktur.

[21] Weil HL, Schneider K, Tschöpe M, Bauer J, Maus O, Frey K (2023). PLANTdataHUB: a collaborative platform for continuous FAIR data sharing in plant research. Plant J.

[22] Regev A, Teichmann SA, Lander ES, Amit I, Benoist C, Birney E (2017). The Human Cell Atlas. Elife.

[23] Leinonen R, Sugawara H, Shumway M (2010). The Sequence Read Archive. Nucleic Acids Res.

[24] Clough E, Barrett T, Wilhite SE, Ledoux P, Evangelista C, Kim IF (2023). NCBI GEO: archive for gene expression and epigenomics data sets: 23-year update. Nucleic Acids Res.

[25] O’Cathail C, Ahamed A, Burgin J, Cummins C, Devaraj R, Gueye K (2025). The European Nucleotide Archive in 2024. Nucleic Acids Res.

[26] Instructions for the submission of data and metadata to hca for atlas assembly.

[27] Dörpholz H, Saarenpää S, Ishaque N, Carsanaro S, Križnik M (2024). Ontologies for single-cell experiments.

[28] Birnbaum KD, Otegui MS, Bailey-Serres J, Rhee SY (2021). The Plant Cell Atlas: focusing new technologies on the kingdom that nourishes the planet. Plant Physiol.

[29] DataPLANT Annotated Research Context Specification v3.0-draft.2 (3.0.0-draft.2). Zenodo.

[30] Sansone SA, Rocca-Serra P, Gonzalez-Beltran A, Johnson D, Community ISA (2016). ISA Model and Serialization Specifications 1.0.

[31] DataPLANT ARCs are FAIR Digital Objects. ARC-RDM. ..

[32] Soiland-Reyes S, Sefton P, Crosas M, Castro LJ, Coppens F, Fernández JM (2022). Packaging research artefacts with RO-Crate. Data Sci.

[33] ..

[34] RO-Create Community. RO-Crate profiles. RO-Crate. ..

[35] Crusoe MR, Abeln S, Iosup A, Amstutz P, Chilton J, Tijanić N (2022). Methods included: standardizing computational reuse and portability with the common workflow language. Commun ACM.

[36] ..

[37] Mühlhaus T, Brilhaus D, Tschöpe M, Maus O, Grüning B, Garth C, Heuveline V, Bisheh N (2022). E-Science Tage 2021: Share Your Research Data [Internet].

[38] Demesa-Arevalo E, Dörpholz H, Vardanega I, Maika JE, Pineda-Valentino I, Eggels S (2026). Imputation integrates single-cell and spatial gene expression data to resolve transcriptional networks in barley shoot meristem development. Nat. Plants.

[39] Bolger M, Schwacke R, Usadel B (2021). MapMan visualization of RNA-seq data using Mercator4 functional annotations. In: Dobnik D, Gruden K, Ramšak, Ž, Coll A, editors. Solanum tuberosum. Methods in Molecular Biology.

[40] Bandrowski A, Brinkman R, Brochhausen M, Brush MH, Bug B, Chibucos MC (2016). The Ontology for Biomedical Investigations. PLoS One.

[41] Federhen S (2011). The NCBI Taxonomy database. Nucleic Acids Res.

[42] Fragoso G, de Coronado S, Haber M, Hartel F, Wright L (2004). Overview and utilization of the NCI Thesaurus. Comp Funct Genom.

[43] Walls RL, Athreya B, Cooper L, Elser J, Gandolfo MA, Jaiswal P (2012). Ontologies as integrative tools for plant science. Am J Bot.

[44] Cooper L, Walls RL, Elser J, Gandolfo MA, Stevenson DW, Smith B (2012). The plant ontology as a tool for comparative plant anatomy and genomic analyses. Plant Cell Physiol.

[45] Abeyruwan S, Vempati UD, Küçük-McGinty H, Visser U, Koleti A, Mir A (2014). Evolving BioAssay Ontology (BAO): modularization, integration and applications. J Biomed Semant.

[46] The Genomic Epidemiology Ontology.

[47] Dumschott K, Dörpholz H, Laporte MA, Brilhaus D, Schrader A, Usadel B (2023). Ontologies for increasing the FAIRness of plant research data. Front Plant Sci.

[48] Hao Y, Hao S, Andersen-Nissen E, Mauck WM, Zheng S, Butler A (2021). Integrated analysis of multimodal single-cell data. Cell.

[49] Association EOSC ..

[50] Krumsieck J, Leidel A, König P, Harald VONW Github – fairagro/m4.4_sciwin_client: sciwin client: reproducible computational workflows made easy!.

[51] Specka X, Martini D, Weiland C, Arend D, Asseng S, Boehm F (2023). FAIRagro: ein Konsortium in der Nationalen Forschungsdateninfrastruktur (NFDI) für Forschungsdaten in der Agrosystemforschung. Informatik-Spektrum.

[52] Galaxy Community (2024). The Galaxy platform for accessible, reproducible, and collaborative data analyses: 2024 update. Nucleic Acids Res.

[53] Di Tommaso P, Chatzou M, Floden EW, Barja PP, Palumbo E, Notredame C (2017). Nextflow enables reproducible computational workflows. Nat Biotechnol.

[54] Mölder F, Jablonski KP, Letcher B, Hall MB, Tomkins-Tinch CH, Sochat V (2021). Sustainable data analysis with Snakemake. F1000Research.

